# Muscle Electrical Impedance Properties and Activation Alteration After Functional Electrical Stimulation-Assisted Cycling Training for Chronic Stroke Survivors: A Longitudinal Pilot Study

**DOI:** 10.3389/fneur.2021.746263

**Published:** 2021-12-15

**Authors:** Chengpeng Hu, Tong Wang, Kenry W. C. Leung, Le Li, Raymond Kai-Yu Tong

**Affiliations:** ^1^Department of Biomedical Engineering, The Chinese University of Hong Kong, Hong Kong SAR, China; ^2^Institute of Medical Research, Northwestern Polytechnical University, Xi'an, China

**Keywords:** cycling, electromyography (EMG), electrical impedance, functional electrical stimulation (FES), stroke

## Abstract

Electrical impedance myography (EIM) is a sensitive assessment for neuromuscular diseases to detect muscle inherent properties, whereas surface electromyography (sEMG) is a common technique for monitoring muscle activation. However, the application of EIM in detecting training effects on stroke survivors is relatively few. This study aimed to evaluate the muscle inherent properties and muscle activation alteration after functional electrical stimulation (FES)-assisted cycling training to chronic stroke survivors. Fifteen people with chronic stroke were recruited for 20 sessions of FES-assisted cycling training (40 min/session, 3–5 sessions/week). The periodically stimulated and assessed muscle groups were quadriceps (QC), tibialis anterior (TA), hamstrings (HS), and medial head of gastrocnemius (MG) on the paretic lower extremity. EIM parameters [resistance (R), reactance (X), phase angle (θ), and anisotropy ratio (AR)], clinical scales (Fugl-Meyer Lower Extremity (FMA-LE), Berg Balance Scale (BBS), and 6-min walking test (6MWT)] and sEMG parameters [including root-mean square (RMS) and co-contraction index (CI) value] were collected and computed before and after the training. Linear correlation analysis was conducted between EIM and clinical scales as well as between sEMG and clinical scales. The results showed that motor function of the lower extremity, balance, and walking performance of subjects improved after the training. After training, θ value of TA (*P* = 0.014) and MG (*P* = 0.017) significantly increased, and AR of X (*P* = 0.004) value and AR of θ value (*P* = 0.041) significantly increased on TA. The RMS value of TA decreased (*P* = 0.022) and a significant reduction of CI was revealed on TA/MG muscle pair (*P* < 0.001). Significant correlation was found between EIM and clinical assessments (AR of X value of TA and FMA-LE: *r* = 0.54, *P* = 0.046; X value of TA and BBS score: 0.628, *P* = 0.016), and between sEMG and clinical scores (RMS of TA and BBS score: *r* = −0.582, *P* = 0.029). This study demonstrated that FES-assisted cycling training improved lower limb function by developing coordinated muscle activation and facilitating an orderly myofiber arrangement. The current study also indicated that EIM can jointly evaluate lower extremity function alteration with sEMG after rehabilitation training.

**Clinical Trail Registration:** The study was registered on the Clinical Trial Registry (trial registration number: NCT 03208439, https://clinicaltrials.gov/ct2/show/NCT03208439).

## Introduction

Stroke is a leading cause of disability that impairs sensorimotor and cognitive functions and the activities of daily life (ADL) and contributes to a huge burden on the health, financial, and social resources (1). Gait-related disorders cause significant inconvenience in the ADL of survivors, and persons with stroke meet great challenge to perform smooth ambulation, especially in normal walking and cycling (2). Therefore, a suitable training protocol is desired and essential for them to restore their muscle strength and to regain their motor function (3).

Functional electrical stimulation (FES) and cycling training are common strategies for improving ambulatory function in rehabilitation after stroke. With a positive orthotic effect from FES (4, 5) and symmetrical coordination of muscle contraction during cycling (6), the FES-assisted cycling training was demonstrated in previous studies to bring crucial benefits to chronic stroke survivors by promoting their walking ability (7–11). However, the inherent properties of muscle changes after FES-assisted cycling training are limitedly explored. Besides motor functional deficits, muscle composition and structure significantly alter as stroke proceeds. Those muscle changes include reduction of quantity and proportion of muscle fiber, intramuscular fat infiltration (12), muscle volume, and lean muscle mass dropping (13–15). These muscle changes contribute to a decrease in force production and functional performance of the muscle (16). Therefore, it is necessary to study the muscle composition, structure, and function alterations related to training for stroke survivors, which will reinforce the understanding of the improvement after the intervention.

Electrical impedance myography (EIM) has been considered as a biomarker to assess the neuromuscular disease progression (17) with high repeatability and reliability (18). During EIM assessment, a weak, high-frequency alternating current flows through the muscle that has been evaluated and produces resultant voltage without causing neuron depolarization and muscle contraction (19). The EIM assessment computes electrical impedance parameters of muscles which reflects the inherent properties of the muscle, including resistance (R), reactance (X), phase angle [θ = arctan (X/R)], and anisotropy ratio (AR). These parameters are related to muscle volume conduction properties (VCPs) (20). The VCPs are objective physical properties of muscle that are determined by the presence of extracellular and intracellular fluid and by the integrity of cell membranes and tissue interfaces (21–23). Among the EIM parameters, resistance is defined as the inherent resistivity of the assessed muscle, which is influenced by the intracellular and extracellular fluids and fat. Reactance represents the delay in conduction caused by cell membranes, non-ionic substances, and tissue interfaces. The R and X values might be influenced by muscle shape, mass, and tissue integrity (21). To reduce the impact of muscle size on impedance analysis, the θ value is calculated based on the R and X values, which evaluate membrane oscillation properties of the muscle (23). The AR value represents the degree of columnar order in the arrangement of the fibers (24). Higher AR value means a more regular arrangement of myofibers (24–26). In comparison with other muscle property assessment techniques, such as MIR and electromyography, the EIM requires less cooperation from the patients and has shorter time on performing the measurement (27). Lately, Li et al. applied EIM in assessing muscle changes after stroke and demonstrated significant difference in electrical impedance and anisotropic properties of muscle between the paretic side (side dominated by lesional hemisphere) and the nonparetic side (side dominated by contralesional hemisphere) (28). Previous studies also combined EIM with other techniques to further explore the alteration of muscle inherent properties after stroke, including the assessment of ultrasound (29) and compound muscle action potentials (30). In our previous study, EIM parameters, such as X and θ values, were correlated with the muscle structure characteristics assessed by ultrasound in stroke survivors (29), and this pilot ultrasound-EIM combined study revealed that the feasibility of EIM intuitively evaluates the muscle structure alteration with medical images (29). However, there is limited exploration of the application of EIM for assessing the training effects after stroke.

Surface electromyography (sEMG) is widely applied to evaluate muscle activation and muscle contraction pattern. A root mean square (RMS) value of sEMG signal is commonly calculated, which directly reflected the muscle activation ability (31). In addition, the co-contraction index (CI) is computed between two related muscles to identify the muscle contraction pattern during movements (32). The aim of this study was to apply EIM and sEMG to track the muscle inherent properties and activation changes of paretic lower extremity muscles after FES-assisted cycling training for chronic stroke survivors. We hypothesized that muscle impedance properties of paretic muscles would change toward those of the non-paretic muscles, and muscle contraction coordination would also improve after training, and those EIM and sEMG changes might be related to the improvement of clinical scales. The findings of this study could provide insights for clinical evaluation of training effect in muscle weakness and functional deficits on chronic stroke survivors.

## Methods

### Participants and Study Design

Subjects were recruited from primary rehabilitation centers in Hong Kong. After recruitment, they were invited to the lab on the campus for the training. The inclusion criteria were as follows: (1) diagnosed as stroke with hemiplegia; (2) more than 6 months duration after the onset; (3) significant gait deficit (Functional Ambulatory Category, FAC < 4). The exclusion criteria were as follows: (1) any additional medical or psychological condition that would affect their ability to comply with the study protocol, e.g., a significant orthopedic or chronic pain condition, major poststroke depression, epilepsy, and artificial cardiac pacemaker/joint; (2) severe hip, knee, or ankle contracture that would preclude passive range of motion of the leg. All of the subjects gave written consent before the experimental procedures. This study was approved by the Joint Chinese University of Hong Kong-New Territories East Cluster (CUHK-NTEC) Clinical Research Ethics Committee (Ref. no: 2016.093), and all the procedures of this study have been operated in accordance with the Declaration of Helsinki. This study was registered on ClinicalTrials.gov (NCT 03208439). This study utilized a self-controlled design with the combination of clinical scales, EIM, and sEMG assessments on lower extremities after FES-assisted cycling training.

### Apparatus and Procedures

#### FES-Assisted Cycling Training

**Figure 1:**
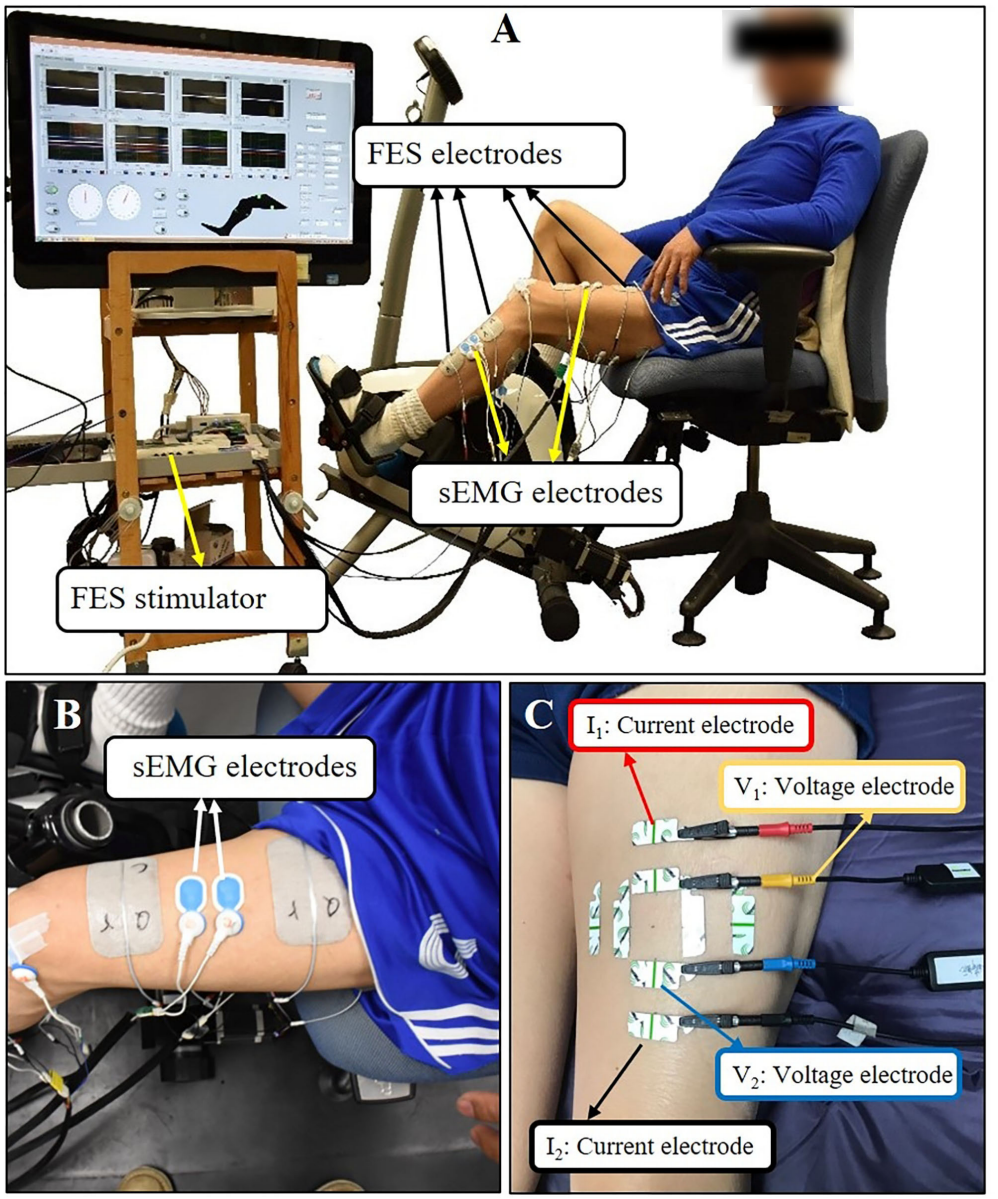
**(A)** The setup of FES-assisted cycling training system. The pedaling angle was monitored by a goniometer. **(B)** Using quadriceps as an example for sEMG electrodes placement. **(C)** Using quadriceps as an example for EIM electrodes placement. The outer two electrodes (the red and black one) were current electrodes, inner two electrodes (the yellow and blue one) were voltage electrodes. Each test lasted for few seconds and three tests were conducted repeatedly at each direction of the arrangement.

**Figure 2:**
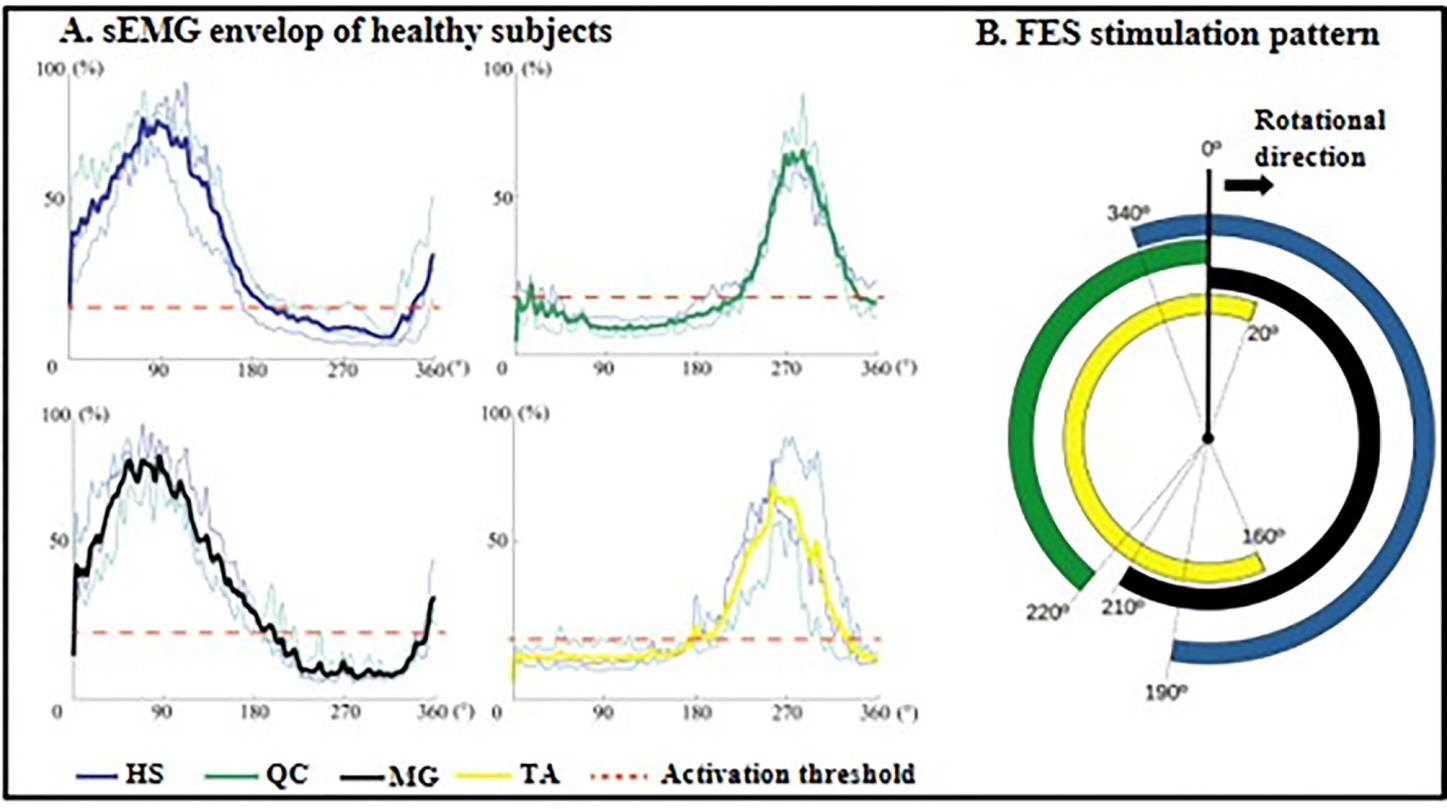
FES stimulation ranges generated from healthy subjects. **(A)** Mean sEMG envelop of three normal subjects: HS, dark blue; QC, dark green; GL, black; TA, yellow. The red dotted lines are the muscle activation thresholds. The y-axis is the contraction level in the range of 0 to 100% normalized by MVC. **(B)** FES stimulation pattern within one revolution: four averaged signals mapping periods of stimulation during cycling. HS, hamstrings; QC, quadriceps; MG, medial head of gastrocnemius; TA, tibialis anterior.

The cycling equipment includes a pair of pedal meters (PowerTap P1 Pedal Meter, USA) and motor (UIRobot UIM241Co4P-IE/57-76, China). During the cycling training, the subjects were seated on a chair of the cycling system with standardized height of seat and pedaled with voluntary muscle contraction under the assistance of FES. A goniometer was used to measure the angle of pedals (Figure 1A). A portable four-channel programmable FES device (Easy Walker, P2-9632 Fine Cure, China) including FES surface electrodes (AXLEGAARD PALS electrodes; Axelgaard Manufacturing Company, CA, USA) were applied. A programmed interface running on the Labview platform was displayed on a computer screen which was viewed by the subjects. Four pairs of electrodes were attached on four paretic lower limb muscle groups and each pair of electrodes was linearly arranged symmetrically on the two sides of sEMG electrodes with a 1 cm interval distance between two neighboring electrodes (Figure 1A). For quadriceps (QC), medial head of gastrocnemius (MG), and hamstrings (HS), 5 cm ^*^ 8 cm surface electrodes were used, and the 5 cm^*^ 5 cm surface electrodes were also used for tibialis anterior (TA). The parameters for FES were set at a pulse width of 300 μs and the stimulation frequency was set at 20 Hz (8). The FES stimulation pattern was periodically constructed from muscular activation intervals identified on three healthy volunteers (male, 24 ± 2-year-old) during pedaling. The ranges, where FES stimulation happens, were mapped with the muscle activation of healthy subjects in one revolution as illustrated in Figure 2. The FES stimulation would be turned “ON” in 340°−190°, 220°−360°, 0°−210°, and 160°−20° on HS, QC, MG, and TA respectively. Every recruited subject received 20 sessions of training (3–5 sessions/week). For every session, firstly, the skin of QC, HS, TA, and MG muscles was exfoliated and cleaned before attaching the electrodes and then, the stimulation intensity was set. The stimulation intensity could be adjusted between 0 and 60 mA and the ideal stimulation intensity produced a visible good contraction of the corresponding muscle to induce a joint movement without any pain and discomfort.

Before pedaling, the angle of the pedals was calibrated, and the 0° of the pedal was set so that the two pedals were in the same line which was vertical to the ground. In each training session, 2 trials were performed and each of them lasted 10 min with a 5-min break between the trials. Each trial included a 2-min warm-up period of voluntary cycling at a cadence of 15 rpm, a 7-min training period of cycling-assisted by FES, and a 1-min cool down period of passive cycling. During passive cycling, the legs of the subjects were passively moved by a motor at a cadence of 15 rpm. Subjects were instructed to normally pedal with their own efforts in the warm-up and training period. One cycling training session took ~40 min.

#### Surface Electromyography Assessment

The sEMG parameters were computed to evaluate muscle activation. At first, skin preparation was conducted by using an alcohol pad. Four pairs of sEMG electrodes (Ambu BlueSensor N Electrode, Denmark) were attached parallelly to the muscle fascicles on the paretic muscle groups (Figure 1B). An EMG amplifier developed in-house was placed closer to each set of EMG electrodes in order to minimize the amount of motion artifacts picked up and amplified during cycling. The in-house amplifiers have a gain of 1,000 at 3dB, at an input impedance of 10 GΩ. The sEMG baseline and maximum voluntary contraction (MVC) measurements of each muscle group were conducted before pedaling. When performing MVC, the subjects were sitting on the chair of the cycling system and were instructed to fully extend the knee joint for QC MVC, to fully flex the knee joint for HS MVC, to fully dorsiflex the ankle joint for TA MVC, and to fully plantarflex the ankle joint for MG MVC. For the QC and HS MVC measurements, the subjects were instructed to keep flex knee joint at 90° and hip joint at 80°. For the TA and MG MVC measurements, the subjects were instructed to keep knee joint at 160° and to keep the ankle joint at a neutral position. Each measurement lasted for 4 sec, and for each muscle, the MVC was measured twice. Then, the subjects were instructed to conduct pedaling, and at the same time, sEMG recording started. We mainly used the sEMG signal from the constant speed pedaling of the first 2 min (warm-up cycling phase) of the first and the last training sessions for data analysis. The sEMG data were collected at a 2,000 Hz sampling rate. The following standard signal processing procedures were applied: (1) power line artifact removal; (2) stimulus artifact removal; (3) cubic spline interpolation; (4) bandpass filtering with cut off frequencies at 20 Hz and 450 Hz (3-order Butterworth filer); (4) average signal subtraction; (5) full-wave rectification; (6) lowpass filtering (3-order, 5Hz Butterworth filer); (7) signal normalization according to MVC. After performing the above seven steps, the envelope of sEMG in each revolution was generated. In order to illustrate the sEMG-angle results clearly, resampling was done for sEMG to obtain the uniform data size for all parameters in every revolution (10,000 sEMG data points in one revolution). After performing sEGM signal processing and resampling, we acquired angle-related sEMG data from different revolutions; then, we calculated the parameters in each revolution (excluded the first five revolutions) and averaged the parameters from all revolutions for comparison.

#### Electrical Impedance Myography

Electrical impedance myography (Imp SFB7 Impedimed, Inc., Sydney, NSW, Australia) measurements were performed before the first training and after the last training session on bilateral interest muscle groups. Subjects were instructed to fully relax and lie in supine (for QC and TA) and prone (for HS and MG) positions. The location of the electrodes for each muscle group was marked as a dot: (1) TA: the proximal one-third distance from the proximal fibular head to the center of the medial malleolus; (2) MG: the proximal one-third distance from the medial aspect of the popliteal fossa to the heel; (3) HS: the distal one-third distance from the medial of the popliteal fossa to the ischial tuberosity; (4) QC: the distal one-third distance from the anterior superior iliac spine to the superior border of the patella.

Saline was applied to clean the skin before each test. For the EIM assessment, four electrodes (one pair of voltage electrodes on the inner regions and an outer pair of current electrodes) were linearly arranged parallel to the direction of the myofiber arrangement. Another two pairs of electrodes were linearly arranged perpendicular to the direction of the myofiber arrangement (33). The center-to-center distance of the inner two voltage electrodes was 30 mm and that of the outer two current electrodes distance was 80 mm (34). Each pair of electrodes was distributed symmetrically along the dot that was marked in advance. The dimension of the electrodes was 2.5 cm^*^ 1 cm (Figure 1C). Every target muscle was assessed three times at parallel and perpendicular directions, respectively; then the average value of the data was computed for statistical analysis.

#### Clinical Assessments

The 6-min walk test (6MWT), the Fugl-Meyer Assessment of Lower Extremity (FMA-LE) [FMA for ankle joint and coordination (FMAac), the FMA for knee joint and coordination (FMAkc)], and the Berg Balance Scale (BBS) were carried out to evaluate the ambulation function of the participants, lower limb motor functions, and the balance before and after the training. All these assessments were carried out by a fixed professional physical therapist who was blinded to the training as well as to the EIM and sEMG results.

### Data Processing

All the variables of EIM and sEMG were exported for offline analysis. Raw impedance variables were R, X, and θ values across multiple frequencies of alternating current from 5 to 1,000 kHz. Raw EIM data collected at the frequency of 50 kHz was analyzed and compared (28) because the frequency of 50 kHz has been widely used as a standard for single-frequency analysis in EIM (35). The AR of R, X, and θ (AR of R, X, and θ) were computed based on the raw variables.

The AR of X, R, and θ was computed by the following Equation (1):


(1) $$\begin{array}{l} AR_{V}=\frac{V_{Perp}}{V_{Para}} \end{array}$$

where V represents the X, R, and θ values, respectively; *V*_*Perp*_ and *V*_*Para*_ represent these variables collected from perpendicularly and parallelly arranged electrodes, respectively.

The RMS and CI were used to characterize muscle activation and coordination.

We calculated the normalized RMS value by Equation (2):


(2) $$\begin{array}{l} RMS_{i}=\sqrt{\frac{1}{T}\int_{0}^{T}{\left[EMG_{i}\left(t\right)\right]}^{2}dt} \end{array}$$

Where *i* is the assessed muscle, including QC, HS, TA, and MG. *RMS*_*i*_ is the normalized RMS value of muscle *i* in one revolution. T is the length of the signal. A decrease in the RMS value represented the improvement of muscle activation volume, which means the subjects can perform the same task with lower efforts.

The CI value was calculated by the following Equation (3):


(3) $$\begin{array}{l} CI_{ij}=\;\frac{1}{T}\int_{0}^{T}A_{ij}\left(t\right)dt \end{array}$$

Where *A*_*ij*_ is the overlapping area of sEMG linear envelops for the muscles *i* and *j* in the warm-up cycling phase, and T is the length of the signal. The CI value varied from 0 (non-overlapping of the muscle pair activation in a specific movement) to 1 (the two muscles' activation totally overlapped with both normalized sEMG activation levels kept at 1 during the movement) (36). The reduction of CI after intervention revealed a more coordinated muscle contraction pair during cycling. Only CI values of agonist and antagonist muscle pairs (TA and MG, QC and HS) were calculated in the current study, to precisely analyze muscle coordination within one joint movement.

### Statistical Analysis

The clinical scores (FMALE, FMAac, FMAkc, 6MWT, and BBS), EIM variables (AR, R, X, and θ), and sEMG parameters (CI and RMS) were reported using mean and standard error. The “1+” score in MAS was coded as “1.5” for statistics (37, 38). Comparisons of EIM parameters between paretic and nonparetic sides as well as before and after all the training were conducted by the paired *t*-test if the data set was normally distributed. Comparison of clinical scores and sEMG variables between pretraining and posttraining was conducted by the paired *t*-test for the normally distributed data set. Data normality was verified by the Shapiro–Wilk test. Wilcoxon signed ranks test was performed for non-normally distributed data set (28). Pearson correlation was conducted on EIM, sEMG, and clinical scores if the data sets were normally distributed; otherwise, the Spearman correlation was applied. Statistical significance was defined as P <0.05, two-tailed for all calculations. Statistical analysis was conducted by SPSS23 (IBM Inc., WA, USA).

## Results

Fifteen subjects with hemispheric stroke (9 women and 6 men, aged 58.1 ± 10.1 years, duration 1–28 years) were recruited in this study (Table 1). All subjects claimed no history of taking anti-spastic medication half-a-year before the training and during the training. One subject (a 40-year-old woman) dropped out from the training after 11 sessions because of personal reasons. We conducted a clinical assessment on this subject after 11 training sessions; however, the sEMG signal was not recorded; therefore, an intention to treat was conducted to do the statistical analysis. Table 1 shows the demographic and clinical information of the cohort.

**Table 1:** Clinical information of subjects.

**ID**	**Gender**	**Paretic side**	**Stroke type**	**Age (year)**	**Duration (year)**	FEMALE^*^		BBS^*^		6MWT(m)^*^		**MAS (HS/QC)**		**MAS (TA/MG)**	
						**Pre**	**Post**	**Pre**	**Post**	**Pre**	**Post**	**Pre**	**Post**	**Pre**	**Post**
1	Female	L	I	54	14	24	26	54	53	252	286	0/1	0/0	0/1	0/1
2	Female	L	H	58	2	30	29	50	51	260	331	1/1	1/1	1/1	1/1
3	Female	L	I	68	9	25	26	49	50	224	247	1.5/2	1.5/1.5	1.5/1.5	1.5/1.5
4	Male	L	H	51	1	7	14	14	14	32	29	1/1.5	0/0	0/1	1/0
5	Female	R	H	59	6	24	26	54	55	304	293	1.5/1	1/1	1/2	2/1
6	Male	R	I	37	4	25	25	53	54	295	315	1.5/1	1/1	1/2	1/2
7	Male	R	H	55	6	16	18	50	50	245	278	1.5/1.5	1.5/1.5	1.5/2	1/1.5
8	Female	L	I	72	4	21	28	49	53	173	210	1/1	1/1	1/1	0/0
9	Female	L	I	72	5	17	18	40	45	59	64	1/1	1/1	1/1.5	1.5/1.5
10	Male	L	I	63	2	25	22	56	56	170	127	1/1	1/1	1.5/2	1.5/1.5
11	Male	L	I	57	2	28	29	55	55	366	432	1/1	1/1	1/1	1/1
12	Female	R	U^□^	40	28	21	28	55	56	340	450	1.5/1.5	1.5/1.5	1.5/2	1/1
13	Male	L	I	61	10	24	23	53	53	192	232	1/1	1.5/1.5	1.5/0	1/1
14	Female	R	I	65	4	16	21	42	51	185	205	1/1	1/1	1/1	1/1
15	Female	R	I	60	3	20	25	55	56	264	323	1.5/1.5	1.5/1	1.5/1.5	1/1
Mean				58.1	6.67	21.5	23.9	48.6	50.1	224	255	1.13/1.20	1.03/1.00	1.07/1.37	1.03/1.07
SE				2.6	1.8	1.5	1.6	2.7	2.6	24.1	25.5	0.10/0.08	0.12/0.12	0.13/0.15	0.13/0.14

*FML-LE, Fugl-Meyer Assessment of Lower Extremity; Pre, pretraining; Post, post-training; L, left; R, right; I, ischemic; H, hemorrhagic; U, unknown*.

* *Significant difference between pre and posttraining*.

□ *This subject suffered from paralysis due to stroke; however, she forgot the type of stroke and the diagnose recording was missing, and she dropped out of the trial after 11 sessions of the training because of personal reasons*.

### EIM Parameters

**Figure 3:**
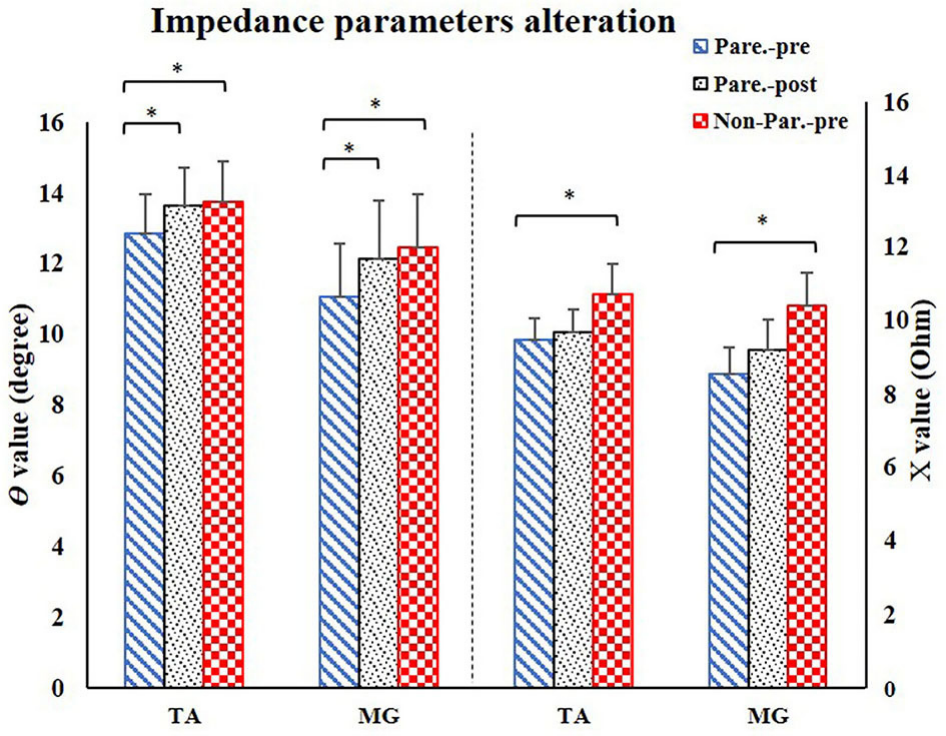
EIM parameters alteration after training. For comparison between paretic and non-paretic muscles, there is a significant reduction of θ and X value in paretic TA and MG. After training, the paretic TA and MG both presented significant increase of θ value. TA, tibialis anterior; MG, medial head of gastrocnemius; X, reactance; θ, phase angle. ^*^P < 0.05.

**Figure 4:**
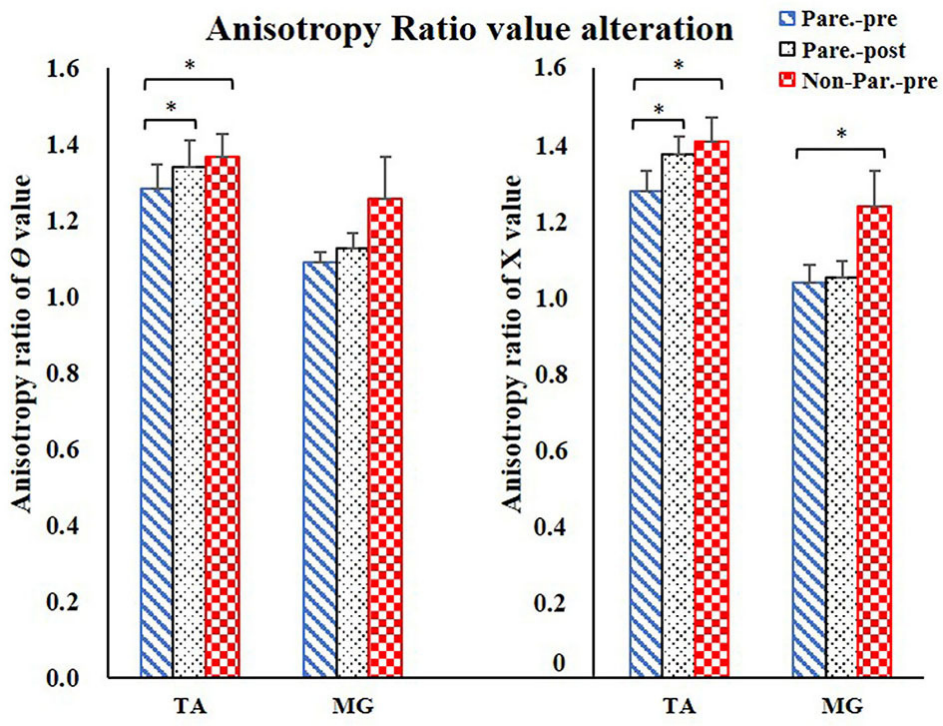
Anisotropy Ratio parameter comparison. For comparison between paretic and non-paretic muscles, significantly lower AR of θ value was shown in TA, significantly lower AR of X value was shown in TA and MG. After training, only TA presented significant increase on the AR of θ and AR of X values. AR, anisotropy ratio; X, reactance; θ, phase angle; TA, tibialis anterior; MG, Medial head of gastrocnemius. ^*^P < 0.05.

The EIM variables of R, X, θ, and AR of four measured muscle groups were compared between the paretic and non-paretic sides. The θ value of paretic muscles were significantly lower than that of the non-paretic side (QC: *P* = 0.004, TA: *P* = 0.009, HS: *P* = 0.002, MG: *P* = 0.004). The X values were significantly lower in the paretic muscles of QC (*P* < 0.001), TA (*P* = 0.007), and MG (*P* = 0.002). The R values were significantly lower in the paretic muscles including TA (*P* = 0.014) and MG (*P* = 0.027) (Table 2). After 20 training sessions, statistical results revealed that the θ value significantly increased in paretic TA (*P* = 0.014, pretraining: 12.6 ± 1.22, post-training: 13.64 ± 1.08), and MG (*P* = 0.017, pretraining: 11.04 ± 1.56, posttraining: 12.14 ± 1.68) (Figure 3). AR value comparison showed that only paretic TA presented a significant increase in the AR of θ value (*P* = 0.041, pretraining: 1.283 ± 0.062, posttraining: 1.339 ± 0.069) and AR of X value (*P* = 0.004, pretraining: 1.275 ± 0.054, posttraining: 1.374 ± 0.046) after training (Figure 4). QC and HS showed no significant alterations of EIM parameters after training (*P* > 0.05).

**Table 2:** Comparison of EIM parameters before training.

**Muscle**	**EIM parameters**	**Paretic side (mean ± se)**	**Non-paretic side (mean ± se)**
QC	R	76.93 ± 6.73	75.00 ± 6.38
	X^*^	8.74 ± 0.74	10.21 ± 0.66
	θ^*^	7.42 ± 1.17	8.69 ± 1.15
TA	R^*^	44.39 ± 2.88	47.99 ± 2.8
	X^*^	9.83 ± 0.62	11.11 ± 0.88
	θ^*^	12.83 ± 1.12	13.73 ± 1.14
HS	R	64.01 ± 5.83	61.10 ± 5.39
	X^*^	8.30 ± 0.52	8.71 ± 0.51
	θ^*^	8.54 ± 1.2	9.34 ± 1.24
MG	R^*^	50.79 ± 4.47	54.55 ± 4.85
	X^*^	8.86 ± 0.76	10.79 ± 0.95
	θ^*^	11.04 ± 1.5	12.46 ± 1.5

* *Significant difference of EIM parameters between paretic and non-paretic sides. HS, hamstrings; QC, Quadriceps; MG, medial head of gastrocnemius; TA, tibialis anterior*.

### sEMG Parameters

**Figure 5:**
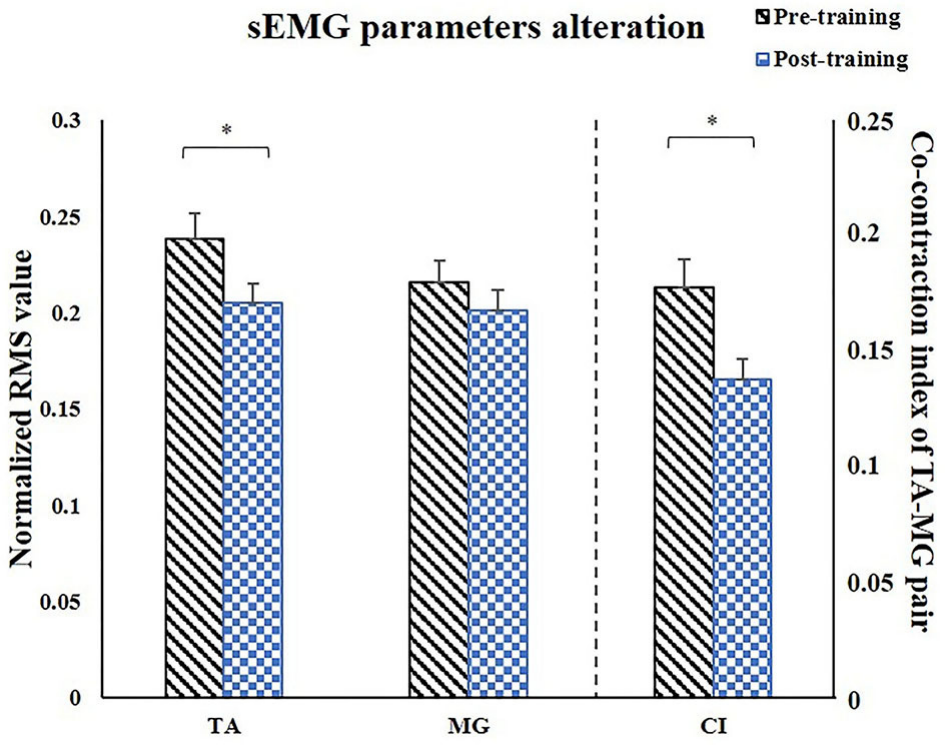
Comparison of the paretic muscles using sEMG parameters. After training, RMS of TA significantly decreased, CI of TA/MG muscle pair significantly decreased. sEMG, surface electromyography; TA, tibialis anterior; MG, medial head of gastrocnemius; CI, co-contraction index; X, reactance; θ, phase angle. ^*^P < 0.05.

**Figure 6:**
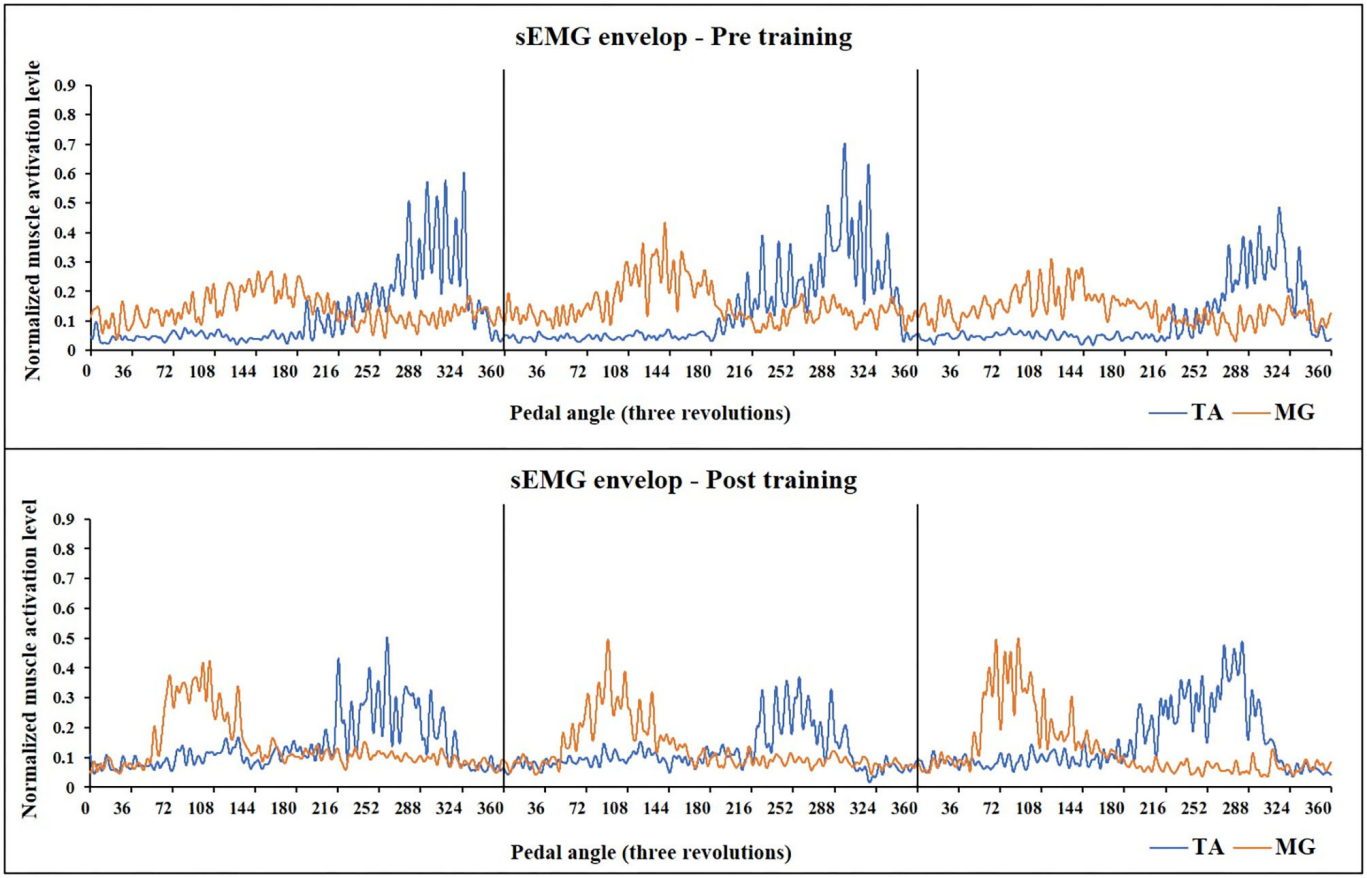
Muscle activation alteration of TA and MG before and after training in one subject (Subject No 12). The sEMG-enveloped signal during the middle three revolutions of cycling is demonstrated. X-axis represents the cycling angles of the pedals. The zero degree of the pedal was set so that the two pedals were in the same line which was vertical to the ground. Yellow curve: sEMG envelope of MG, Blue curve: sEMG envelope of TA. After training, the activation of both two muscle pairs decreased, and MG muscle presented a more independent and rhythmic activation pattern (CI value of TA and MR muscle pair: pretraining: 0.168, posttraining: 0.139). TA, tibialis anterior; sEMG, surface electromyography; MG, medial head of Gastrocnemius.

For sEMG parameters, RMS value of paretic TA significantly decreased after training (*P* = 0.022, pretraining: 0.24 ± 0.01, posttraining: 0.2 ± 0.01). The CI between paretic TA and MG significantly reduced after the training (*P* < 0.001, pr-training: 0.21 ± 0.01, posttraining: 0.16 ± 0.01) (Figure 5). Figure 6 demonstrates muscle contraction pattern alteration during three revolutions of cycling of one recruited subject, in which the contraction of TA and MG was more coordinated after training with less overlapping area under the curves (CI value of TA and MR muscle pair: pretraining: 0.168, posttraining: 0.139) (Figure 6). QC and HS showed no significant alteration of RMS and CI value after training (*P* > 0.05).

### Clinical Assessments and Correlation Analysis

**Figure 7:**
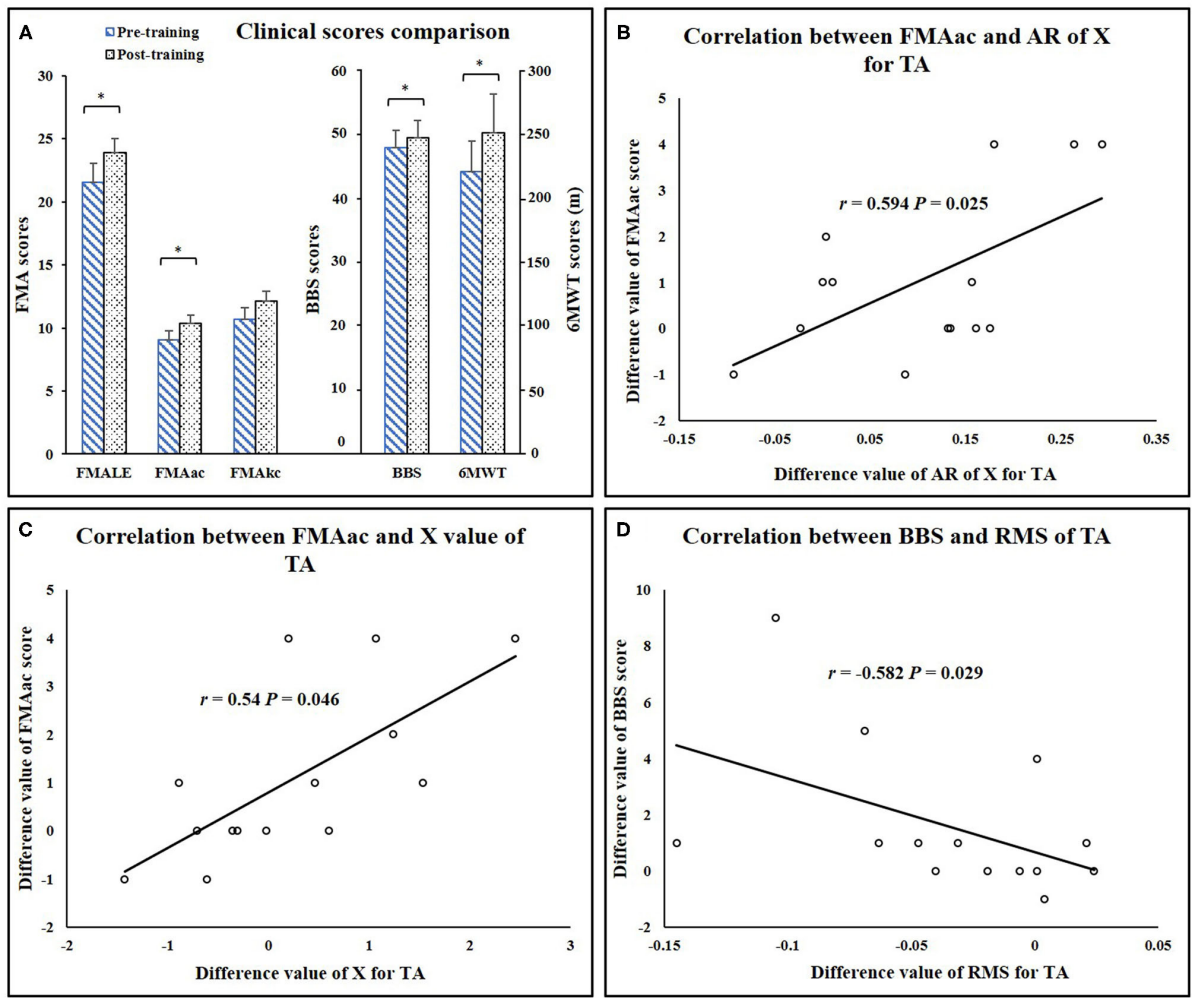
**(A)** Clinical score comparisons. **(B)** Correlation between FMAac and AR of X value for TA. **(C)** Correlation between FMAac and X value of TA. **(D)** Correlation between BBS and RMS value of TA. AR, anisotropy ratio; RMS, root mean square; TA, tibialis anterior; FMA-LE, Fugl-Meyer assessment of lower extremity; FMAac, Fugl-Meyer assessment of ankle joint and coordination segments; FMAkc, Fugl-Meyer assessment of knee joint and coordination segments; BBS, Berg Balance Scale; 6MWT, 6-minute Walking Test. ^*^P < 0.05.

Significant increases in clinical scores (FMA-LE: *P* = 0.013, FMAac: *P* = 0.02, 6MWT: *P* = 0.009, BBS: *P* =0.038) (Table 1) were observed after the training. Mean MAS scores decreased after training, while the differences were not significant (Ps > 0.05). There was no significant alteration of FMAkc score after training (*P* = 0.058) (Figure 7). Significant correlation was demonstrated between EIM parameters and clinical scores, including AR of X value for TA and clinical score (AR-X-TA and FMA-LE score: *r* = 0.54, *P* = 0.046, AR-X-TA and FMAac score: *r* = 0.594, *P* = 0.025), as well as the X value of TA and clinical scores (TA-X and FMAac: *r* = 0.692, *P* = 0.006; TA-X and BBS: *r* = 0.628, *P* = 0.016). Significant correlation was indicated between sEMG and clinical scores, including RMS of TA and BBS score (*r* = −0.582, *P* = 0.029), RMS of MG and FMA-LE (*r* = −0.618, *P* = 0.019), and RMS of MG and FMAac (*r* = −0.653, *P* = 0.011) (Figure 7).

## Discussion

In this study, EIM and sEMG measurements were conducted in stroke survivors before and after FES- assisted cycling training. The application of EIM and sEMG analysis for evaluating the changes in the inherent property of the muscle and the level of muscle activation after FES-assisted cycling training is relatively a novel application. The results demonstrated that after training, muscle activation increased and muscle contraction coordination within ankle joint movements improved. Muscle inherent properties of paretic muscles changed in the non-paretic muscles. Significant correlations were found between EIM and clinical scores as well as between sEMG and clinical scores. The lower extremity motor function improved after training, and the subjects acquired better balance and ambulation performance.

### Bilateral Difference

All paretic muscles presented lower θ values compared to the non-paretic muscles. The X value was reduced only in paretic QC, HS, and TA compared to the non-paretic side. The R value was decreased only in TA and MG. These outcomes were consistent with the previous study by Li et al. which reported that θ can be a more sensitive biomarker than X and R values in assessing the inherent muscle property of Biceps Brachii after stroke (28). We also observed significant lower AR values in paretic TA and MG. Reduced AR of X value and θ value after stroke was related to the loss of muscle fibers, increased fat, and connective tissue infiltration (24, 25). Previous histological studies also revealed muscle atrophy and fat tissue accumulation after stroke (12). This cross-sectional result supported that the EIM assessment could also be applied to muscle composition and structure evaluation of lower extremity muscles in chronic stroke survivors.

### Training Effects From EIM and sEMG Measurements

Previous studies reported that the increase in the CSA of muscle, muscle fiber hypertrophy, and intramuscular fat consumption were considered to be a longstanding effect after stroke rehabilitation intervention. Increased muscle mass and decreased intramuscular fat were related to the improvement of muscle strength (16, 39). Ryan et al. and Chae et al. found that muscle CSA and muscle fiber size increased and the intramuscular fat reduced after FES and cycling training (5, 40). Walls et al. and Herrero et al. found that the muscle fiber size and muscle CSA increased after FES intervention (41, 42). Natsume et al. (43) reported that FES could result in muscle hypertrophy because of recruiting both slow- and fast-twitch muscle fibers (43). Therefore, due to these factors, muscle fiber size increased and myofiber cytomembrane area enlarged, which increased the charge storage capability of the cytomembrane (22). This led to a higher time shift in this study when alternating current passed through the muscle in EIM evaluation, and resulted in the elevation of θ value after training (Figure 3). On the other hand, the AR value of TA increased after training in the current study, which further suggested a more regular myofiber arrangement after training compared to the one before training. In a fixed muscle volume, the increase of muscle fiber size caused the myofibers to arrange more tightly, which improved the characteristic of muscle anisotropy and made the electrical current flow tougher in the direction perpendicular to the fiber than in the longitudinal direction (29). In addition, the isotropic intramuscular fat reduction would increase muscle anisotropy property in return. These two points might be the reason for the increase in AR value after the training (Figure 4).

From a perspective of myofiber structure, ultrasonography was widely applied to evaluate muscle structure alteration after training. Liu et al. used B-mode ultrasound to assess TA and MG architecture alteration after 3 weeks of body weight-supported treadmill training in stroke survivors (44). Significant increases in pennation angle and muscle thickness after the training were reported in FES- assisted lower extremity training (44–47). In our previous cross-sectional study, EIM and ultrasound were jointly applied to explain muscle composition and structure alteration during passive muscle stretch for subacute stroke survivors. In paretic TA, the lower pennation angle and muscle thickness value might contribute to the reduction of the X and θ values compared to the non-paretic TA muscle (29). Therefore, in the current study, the increase of X and θ values on TA could be related to the higher pennation angle and muscle thickness value after FES-assisted cycling training. The elevation of the pennation angle suggested that more muscle fibers and contractile materials are attached to the tendon and contributed to more muscle fibers packed in the same cross-sectional area, which promoted force transfer to the tendon (48). Greater pennation angle and higher muscle thickness contributed to higher AR value because under these conditions, muscle fibers are arranged more perpendicularly, and the current would transversely flow through more muscle fibers in a fixed distance of two voltage electrodes during EIM assessment; ultimately, the AR value increased.

Functional electrical stimulation combined with cycling or gait training was widely used to facilitate gait performance by relieving the subjects from “drop foot” (11, 49–51). It induced coordinated function assistance during ankle dorsi-flexion and reduced the energy consumption for stroke survivors during ambulation. Recently, Rouse revealed that muscle activation is improved in an FES cycling study; however, the performance of the clinical function was not evaluated (52). Previous studies reported that the absolute RMS value of paretic TA during maximum dorsi-flexion increased after FES intervention, which revealed that the capacity of the muscle output increased after training (53). In our study, the decrease of the normalized RMS value of TA after training might be attributed to the increase in maximum activation during MVC or less activation during pedaling, which could both reflect development in the muscle activation capacity of TA (54). In addition, the decrease of normalized RMS after training also suggested reduced excessive muscular activities of TA during pedaling tasks (55, 56). Furthermore, the reduction of excessive muscular activation indicated the development in voluntary motor controls (56). Yan et al. found a significant decrease of co-contraction ratio during dorsiflexion of the paretic ankle joint after FES intervention (without cycling) (51). It was explained and cross-validated that frequently repeated movements, induced by FES in this study, might reinforce network connection patterns. The FES-assisted cycling in our study allowed for continuous repetition of movements during pedaling. The assists from FES simulated and synchronized with normal muscle activation pattern from healthy subjects during cycling. Therefore, this synchronized stimulation and repetitive muscle contraction reminded the subjects how to pedal voluntarily and coordinately. An fMRI study reported that the repetitive FES-induced movements facilitated motor learning according to cortical mechanisms and spinal mechanisms (57), which revealed a more independent control of muscle contraction after training. Our study also demonstrated significant muscle co-contraction reduction within ankle movements during cycling (Figure 4). In addition, as shown in Figure 6, the sEMG enveloped signals of TA and MG were more rhythmic after training, the overlapping area of muscle activation decreased obviously. In Brunnstrom's theory, the reduction of TA/MG co-contraction could induce the separation movements of ankle joint and result in function improvement (58). This also explained our clinical results that the FMALE score increased, and the ambulation performance improved after training.

Our study combined EIM and sEMG techniques to jointly evaluate the FES-assisted cycling training effect. The increase of θ and AR value of TA after training may partly reflect that muscle cross-sectional area and muscle mass increased and might be related to the decrease in the intramuscular fat tissue, and the myofiber is arranged more regularly after training (17, 24, 25). Similarly, the current study proved that muscle activation from sEMG and muscle structure changes from EIM were both improved after the FES-cycling training. These results revealed the alteration of muscle properties after training from different points of view, which help us understand the training effect from different aspects.

### Clinical Consideration of Training Effects

Results of clinical scales indicated improvements in motor functions of lower extremity, gait, and balance after 20 sessions of training (Table 1). The walking distance in 6MWT improved 13.8% and the FMALE score increased 11.2% after training compared to that before training, which was similar to previous studies (59). In the study by Janssen, it was found that after FES-induced cycling training, the BBS score increased from 40 to 44.2 (10%) and 6MWT increased from 160 to 185 (15.7%) (11). However, the improvement of balance function after training was relevantly lower than that of the ambulation performance (6MWT) and motor function (FMALE) in the current study. Because most of the recruited subjects presented high BBS scores before training as shown in Table 1, the ability of balance control was not mainly required during cycling exercise (60). Thus, the subjects in this study presented limited improvement in balance function.

Another interesting result was that all the parameters of EIM and sEMG revealed that TA muscle presented the most significant improvement after training, and it was noticed that FMA scores of ankle segment presented a significant increase while no significant alteration was revealed by the FMA scores of knee segment. This outcome might be due to the higher stimulation intensity applied to the TA muscle as compared to the rest of the muscle groups according to our experiment records (Supplementary Material). This can be reasonable because the reduced innervation of TA was the leading cause of drop foot (61). Our correlation analysis also partly explained this unique improvement on TA. As shown in Figure 7, EIM, sEMG, and clinical scores significantly correlated with each other on TA. Therefore, our results partly reveal that after the training, muscle composition, and structure property of TA change toward the direction of nonparetic side of TA, and myofibers might be arranged more regularly and extra tissues (fat and connective tissues) are reduced (partly indicated by EIM), which might facilitate the increase of muscle activation volume during ankle dorsi-flexion (revealed by sEMG). Those alterations in some way possibly promoted ankle joint motor function and enhanced the walking performance. Based on the results, we may speculate that after the repetitive muscle contraction during FES-assisted cycling, the subjects might learn to coordinately control muscle activation (revealed by CI of sEMG), and this motor relearning procedure allowed subjects to perform better in ambulation and balance assessment.

## Limitations

There were some limitations in the current study which needed cautions to interpreting the results. First, no control group was designed, which might inevitably neglect some substantial information to demonstrate the unique characteristics of FES-assisted cycling. The motor function of the lower extremity of the subjects in the current study was moderate to high, which partly affected the improvement after the training; in the future study, the subject pool should cover stroke survivors with different motor function levels. In addition, EIM changes only indirectly reflect the muscle architecture alteration after the training. In the future study, we may utilize ultrasound as an assessment tool to discuss muscle structural alteration along with EIM changes based on our recent findings on paretic TA muscle of stroke survivors (29).

The current pilot study applied only FES on the paretic side muscle and then EIM and sEMG data were not measured at the nonparetic muscles after training, which might be due to lack of some training effect comparison since the cycling training involved both the lower extremities. In future studies, the EIM and sEMG assessment should also be conducted on the non-paretic muscles to provide a full picture of the training effects. Our study found no significant alterations of impedance properties on HS and MG, which might be due to the thicker subcutaneous fat compared to TA and MG. In future studies, ultrasound can be used to measure the thickness of the subcutaneous fat, which might provide more information about its effect on impedance properties measurement. In the current study, only the motor function assessment of the lower extremity was assessed, while the assessments of ADL is not performed. In the future study, the ADL performance (Barthel Index, etc.) will also be recorded, which will help us understand the training effect. Our study did not analyze frequency domain sEMG data, which might give information about the loss in the muscle fiber firing rate information as well as the fatigue status. In the future study, the frequency domain sEMG will be calculated to provide more information regarding muscle function.

## Conclusion

Our study demonstrated the feasibility to combine EIM and sEMG to assess the muscle inherent properties and activation changes in subjects with chronic stroke after FES-assisted cycling training. The motor function improvement of the lower extremity was related to increased muscle impedance properties (intramuscular fat consumption, more regular myofiber arrangement, etc.), higher muscle activation capacity, and better muscle coordination during ankle joint movements. The TA and MG benefited greatly in the FES-assisted cycling training. This study provided insights for clinical evaluation in muscle weakness, functional deficits, and clinical rehabilitation therapy on stroke survivors.

## Data Availability Statement

The datasets analyzed during the current study are available from the corresponding authors upon reasonable request.

## Ethics Statement

The studies involving human participants were reviewed and approved by the Joint Chinese University of Hong Kong-New Territories East Cluster (CUHK-NTEC) Clinical Research Ethics Committee. The patients/participants provided their written informed consent to participate in this study.

## Author Contributions

CH, RK-YT, and LL conceived and designed the study. CH, TW, and KL performed the experiments. CH and TW wrote the paper. RK-YT and LL reviewed and edited the manuscript. All authors had read and approved the manuscript.

## Funding

This study was supported in part by the National Natural Science Foundation of China (Nos. 31771016 and 32071316), the Guangdong Basic and Applied Basic Research Foundation (No. 2020A1515011356), and the Innovation and Technology Fund (Project No. ITS/276/15) of Hong Kong Special Administrative Region (HKSAR).

## Conflict of Interest

The authors declare that the research was conducted in the absence of any commercial or financial relationships that could be construed as a potential conflict of interest.

## Publisher's Note

All claims expressed in this article are solely those of the authors and do not necessarily represent those of their affiliated organizations, or those of the publisher, the editors and the reviewers. Any product that may be evaluated in this article, or claim that may be made by its manufacturer, is not guaranteed or endorsed by the publisher.
