# Supplementary material for: Muscle Electrical Impedance Properties and Activation Alteration After Functional Electrical Stimulation-Assisted Cycling Training for Chronic Stroke Survivors: A Longitudinal Pilot Study
Source: Front Neurol. 2021 Dec 15;12:746263. doi: 10.3389/fneur.2021.746263 (PMC8716001; doi:10.3389/fneur.2021.746263)
Supplement: Supplementary file 1 [file Data_Sheet_1.PDF]

1 Supplementary Material

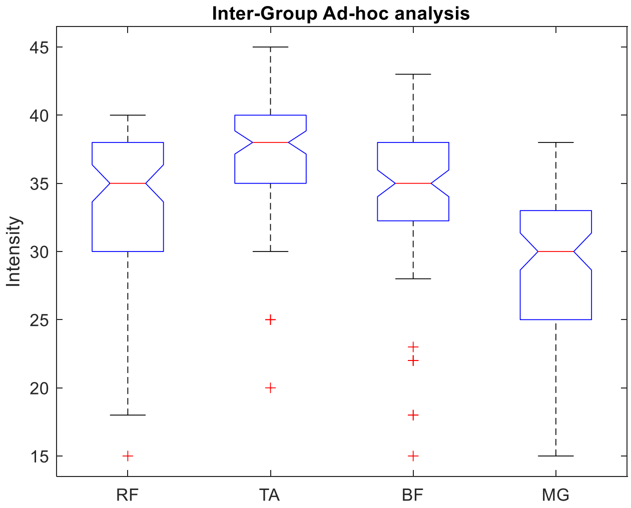

I. Ad-hoc statistical analysis of muscle group stimulation intensity using box-plot showing median and 1st, 3rd quartiles (y-axis corresponds to intensity level as percent of maximum device intensity and x-axis corresponds to muscle groups).
